# In transition with attention deficit hyperactivity disorder (ADHD): children’s services clinicians’ perspectives on the role of information in healthcare transitions for young people with ADHD

**DOI:** 10.1186/s12888-022-03813-6

**Published:** 2022-04-09

**Authors:** Anna Price, Siobhan Mitchell, Astrid Janssens, Helen Eke, Tamsin Ford, Tamsin Newlove-Delgado

**Affiliations:** 1 University of Exeter, St Luke’s Campus, Exeter, EX1 2LU UK; 2 Department of Public Health, University of Southern Denmark, J. B. Winsløws Vej 9B, DK-5000 Odense C, Denmark; 3 Department of Psychiatry, University of Cambridge, Hershel Smith Building, Robinson Way, Cambridge Biomedical Campus, Cambridge, CB2 0SZ UK

**Keywords:** ADHD, Transition, Information, Qualitative, Adolescent, Mental health

## Abstract

**Background:**

National clinical guidelines emphasise the need for good communication of information by clinicians to young people and their parent/carers about what to expect during transition into adult services. Recent research indicates that of young people in need of transition for attention deficit hyperactivity disorder (ADHD), only a minority experience continuity of care into adulthood, with additional concerns about quality of transition. This qualitative analysis explored the role that information plays in the transition from child to adult mental health services for young people with ADHD, from the perspectives of clinicians working in children’s services.

**Methods:**

Participants were recruited from National Health Service (NHS) Trusts located across the United Kingdom (UK), with varying service configurations. Twenty-two qualitative interviews were conducted with 15 paediatricians and seven psychiatrists working in child services and supporting young people with ADHD. The Framework Method was used to complete a thematic analysis of data related to the role of information in transitional care.

**Results:**

Two themes were identified in relation to the role of information in supporting transition and promoting continuity of care. *Information for clinicians;* about adult mental health services, the young person and their ADHD, and exchanged between services. *Sharing information with young people*; about transition processes, self-management, to support service engagement, and tailored to be accessible to young people with ADHD. Clinicians in children’s services reported variable access to information. Clear protocols and being able to communicate about ADHD as a long-term condition, were described as having a positive impact on the transition process.

**Conclusions:**

These findings illustrate that clear information on the transition process, and communication of evidence based and up-to-date information on ADHD as a long-term condition are essential components for clinicians supporting transition into adult services. Information exchange can be supported through transition discussions with young people, and joint meetings between services Discussions should be accompanied by accessible resources for young people and parents/carers such as leaflets and websites. Further efforts should be focussed on enabling clinicians to provide timely and appropriate information to young people with ADHD to support transition.

**Supplementary Information:**

The online version contains supplementary material available at 10.1186/s12888-022-03813-6.

## Background

Attention deficit hyperactivity disorder (ADHD) is a common neurodevelopmental disorder, characterised by inattention, hyperactivity, and impulsivity [4, 48]. Reviews of evidence suggest a worldwide prevalence of 5.3-7.1% in children and adolescents [32, 48], and 2.6-2.8% in adults [15, 44]. As many as two thirds of young people with ADHD continue to experience symptoms into adulthood [2]. A meta-analysis of follow-up studies of children with ADHD found that 15% continue to meet full diagnostic criteria at the age of 25 years, with a further 50% experiencing some degree of continued impairments and subthreshold symptoms [14]. However, in the United Kingdom (UK), the mixed methods “Children and Adolescents with ADHD in Transition between Children’s and Adult Services” (CATCh-uS) study recently reported that very few young people with a clinically confirmed need for ongoing medication for their ADHD made a successful transition to adult services [21]. CATCh-uS was a mixed methods project involving three strands: a surveillance study examining the incidence of need for transition and successful transition; a study mapping adult ADHD services; and a qualitative study exploring the perspectives of young people, parents and clinicians [21].

Transition in health care services is defined as “a purposeful, planned process that addresses the needs of adolescents and young adults with chronic physical and medical conditions as they move from child-centred to adult-orientated health care systems” [7]. Young people with ADHD are one of the groups least likely to make a successful transition to adult mental health services, in what has been described as a ‘failure of healthcare’, complicated by a historical lack of service provision for adults with the condition [12, 41, 42, 51]. This is despite poor outcomes across multiple domains including work and education, relationships, criminal justice, and health, with attendant economic impact among those who prematurely stop their medication [10, 24, 40]. An additional challenge in ADHD relates to stigma and misunderstanding around the condition itself, even on the part of clinicians [16].

For these reasons, transition in ADHD has recently been the focus of concerted research and policy efforts, including the publication of NICE guidance on ADHD with specific recommendations on transition [30]. Multiple barriers and facilitators to healthcare transitions have been identified for young people with long term mental health conditions [13, 27, 31, 34, 45]. A systematic review of qualitative evidence on transition in ADHD reported on hurdles to accessing care (such as meeting referral thresholds), limitations of existing adult mental health services, and inadequate availability of services [34]. Identified facilitators to transition included greater flexibility around age boundaries between services, support from specialist adult ADHD services, and improved provision of information about adult services [34]. The CATCh-uS qualitative strand involved interviews with 144 individual stakeholders from across the UK, including patients, parent/carers, clinicians from child and adult services and general practitioners. The study identified four interrelated factors which could act as barriers or facilitators to continued treatment into adulthood [21]. Firstly, whether ADHD medication was viewed as required only for education, secondly, whether medication was viewed as beneficial, thirdly the extent of parental involvement, and fourthly, feeling prepared for transition and adult life with ADHD [21].

Whilst the qualitative strand in CATCh-uS was not specifically designed to explore the role of information in the process, information-centred themes were prominent in the interviews with young people and parents. Themes identified focused on the essential role of the parent/carer as a navigator and translator of information, and defined two specific types of information: about ADHD into adulthood, and about the transition itself [35].

Access to clear information is a central component of the provision of care as well as self-management for people with long-term conditions, and is essential for both patients and clinicians. Patients need information to maximise their engagement with services and to make the best use of their involvement with services, and clinicians need the relevant patient information and resources to ensure effective clinical management [1, 47]. There are certain junctures at which information in various forms can be particularly crucial. One of these is during the transition from child to adult health services, which often coincides with transitions in education, family life and identity. National Institute for Health and Care Excellent (NICE) guidance on transition [29] recommends that information is provided in a developmentally appropriate format to young people and their parents/carers about available support and adult services; also that information about the young person is communicated between services. Relevant and clear information can facilitate engagement with care, reduce uncertainty, and assist self-management and planning, at a stage when young people may be more prone to drifting away from services [5, 41–43]. Whilst accounts of transition from the patients’ perspective (as discussed above) describe how the clinician is a key source of information in the transition process, little is known about how clinicians view the role of information in transition, their role as information-provider, and their own need for information.

This paper draws on an additional analysis of the CATCh-uS interviews with clinicians in children’s services (Paediatric or Child and Adolescent Mental Health Services (CAMHS)), hereafter referred to as ‘child/children’s services’, about their perspectives on the role of information in facilitating or impeding young people’s transition to adult services (Specialist or Generic Adult Mental Health Services (AMHS)). The full findings of CATCh-uS, including an overview of facilitators of smooth transition are detailed in our report to the NIHR [21]. The aim of this paper is specifically to explore the perspectives of these clinicians on the roles of information in service transition for ADHD and how it affects the success of the process, and influences outcomes for young people, and also to provide recommendations for how information available to clinicians could be improved.

## Methods

This paper presents a focused study on the role of information as reported by clinicians working in children’s services, drawn from the large qualitative study of stakeholder experiences of the transition process in the CATCh-uS study.

### Sampling and recruitment

Practitioners who took part in the CATCh-uS surveillance study and indicated that they were willing to take part in an interview about transition, were purposively sampled. Participants were from NHS children’s services located in different geographical areas of the UK and represented a range of local adult ADHD service models (dedicated ADHD vs. generic Adult Mental Health). For full details of recruitment strategy please see the CATCh-uS report [21].

We anticipated that 20-25 interviews would be needed from each stakeholder group to reach data saturation. A mid-recruitment break (after approximately two-thirds of interviews were completed) was included to assess saturation level, identify novel themes and re-focus the sampling strategy if required. Decisions about sample size drew on experiences of previous studies on transition and wider methodological findings regarding the anticipated stage in data collection when data saturation is likely to occur [6, 26].

### Interview procedure

All participants provided written informed consent, including for audio-recording of the interviews. Semi-structured interviews took place between October 2017 and November 2018, by telephone in order to increase flexibility and accessibility. All interviews were digitally voice-recorded and transcribed verbatim. Each recruited participant was assigned a unique identifier code. Interviews were on average 40 min in duration.

The topic guides for the interviews were developed using the study’s qualitative strand research questions and covered practicalities related to transition, key elements of optimal transition, involvement of families and other services, and personal experience of failed, good and difficult transitions. Questions included “Do you feel you have been able to help the young person to make a sensible decision about what to do after this service/care stops?” and “Have you encountered cases where transition has been particularly difficult? What have been the issues? What steps were taken to resolve the difficulties?” Full topic guides are provided in the CATCh-uS report [21].

### Data management and analysis

Interview recordings and transcriptions were stored on an encrypted hard drive. Once transcribed, interview data were managed using QSR International’s NVivo12 qualitative data analysis software [36] and were stored securely and password protected.

For full details and findings of the initial analysis please see the CATCh-uS report [21]. A recurrent finding identified in this analysis was the significance placed by participants on information (or the lack thereof); both by young people and parents [35], as well as clinicians. Matrix summaries relating to information during the transition process were extracted and analysed further by AP. The first stage of analysis, completed by AP, started with ‘indexing’ a small sample of interviews, to gather an insight and overview of the data. A thematic framework was then created, building on relevant sections of the framework created for the main CATCh-uS report, that identified key concepts and was used to code all remaining interview data [17]. The next stage involved writing summaries of each interview for every code. This allowed for comparison, exploration and explanation of patterns emerging [38]. This method facilitates systematic and transparent data analysis, and enables researchers to identify patterns or commonalities, as well as contradictions in and between participants’ accounts, so they can explore and test explanations for those patterns [39]. Refined themes were reviewed by SM and TND, and final themes and subthemes were then agreed [8]. See Tables 1 and 2.

**Table 1:** Theme 1. Information for clinicians

Sub-theme	Summary	Illustrative quotes (^**a**^)
**About AMHS**	**Which service:** the majority of clinicians had limited or no information about where to transfer young people to. When transition pathways, including names and contacts, were available, it made supporting transition easier.	“*There’s supposed to be a kind of transition path that we could give to young people...we haven’t managed to get that yet and we kind of feel that we can’t move forward with it.” (Child Psychiatrist 13)* *“We now have a psychiatrist who… specialises in adult ADHD, so we now refer to her. So that makes life a lot easier and means that it’s much more straightforward.” (Child Psychiatrist 01)*
	**Referral criteria**: Gathering and preparing accurate referral information was often complex, due to unclear AMHS acceptance criteria, and variation in service availability.	“*I have no idea what their criteria are or whether they have changed...I don’t know the rationale behind that.” (Paediatrician 02)* *“If they have got autism and ADHD and they aren’t on medication it can get really messy... Because there’s no pathway.” (Paediatrician 11)*
	**Transition pathways:** Clinicians were often not familiar with national guidelines on transition; or, if they were, described them as not being specific enough. Local protocols (where available) provided more specific and helpful information, however most clinicians were not aware of an agreed local transition pathway for ADHD, and therefore struggled to know how to facilitate transitions.	*“I generally find them [NICE guidelines] useful. I can’t really recall whether they have anything particularly to say about the transition.”* (*Paediatrician 02)* *“When I first started we didn’t have that and it was a bit more, you know, kind of phoning and looking for people and saying, ‘Are they this team or that?’ So the protocol has massively improved our transition.” (Child Psychiatrist 13)*
	**Transition outcomes**: Information about what happened to patients after transition was not routinely collected, and frequently not available to clinicians in children’s services. Some clinicians did not consider this kind of feedback to be important for transitional care, while the lack of feedback made others uneasy.	*“I would assume many [drop out at transition] but I don't have any knowledge of any. We don't have any effective data monitoring system to know what happens…I will often give them that extra follow-up to make sure that they've come to rest safely. (Paediatrician 18)”* *“I know from the adult services that some of our people we do refer just don’t attend.” (Child Psychiatrist 01)*
**About the young person and their ADHD**	**Young person**: background information about the patient was seen as important to inform transitional care. This was gathered via various methods, including specialist nurses, home visits, conversations with parents, and contacting schools. An automatic reminder (via clinic IT systems) of when patients were approaching transition age, was suggested as a tool to help them to start thinking about transition. One clinician said that when they had only limited background knowledge, this led to poor quality provision of transfer information to AMHS.	*“…keep some sort of intermittent dialogue perhaps through a telephone clinic with the parent just to check all is well, and if all is well then we would discharge them, but if they were still concerned we would tend to get updated information from school in the form of questionnaires …if they look awful then make contact with the parent again and then invite the child to have another chat about life, and if it looks good then we would discharge at that point.” (Paediatrician 16)* *“We could really do with something that flags up a reminder to clinicians to start thinking at 16 about transition or earlier if need be and we don’t have that… Our systems are very primitive.” (Child Psychiatrist 03)* *“Sometimes I’ve only seen them once and then I’m discussing transfer and I’m thinking I hardly know… I don’t have a lot to add to any sort of transfer information. …so I think that’s very frustrating and probably feels rather poor.” (Child Psychiatrist 07)*
	**ADHD as a long-term condition**: understanding and communicating the possible health and social care needs of a young person living with ADHD as a long-term condition was seen as an important, yet challenging, part of supporting transition. Clinicians wanted to be confident in their own knowledge, especially when communicating with patients and their parent/carers. There was variation in how clinicians conceptualised and explained possible health and social needs. Some were vague, some expressed different perspectives on how likely it was that a young person would ‘grow out of’ ADHD, and others provided detailed and nuanced information about what to expect.	*“*…*advising things like driving and other careers and careers that they are excluded from if they are on medication. Sometimes we probably don’t have all the up-to-date information, or have to go away and research it ourselves to make sure that we are not giving the wrong advice.” (Paediatrician 19)* “*…there is also a sense that as young people grow older, that they need it less because they are able to self-manage and understand their strengths and needs...” (Paediatrician 02)* *“I’d say a majority still need it. Some do seem to outgrow their ADHD and that’s great, and I have had that happen, but I’d say a majority still need ongoing management with medication into adulthood.” (Paediatrician 09)* *“…with ADHD, normally the hyperactivity has settled down by then, the impulsivity is there to a certain extent, it's the disorganisation that's a big problem and the emotional dysregulation…” (Paediatrician 05)* *“…there are so many different issues. I suppose what I'm trying to say is that for something like ADHD where the diagnosis has a whole lot of subjective socially bits coming into it … I think that I can give an experienced medical take [to the patient] on … something where there isn't a right or a wrong answer. We can have this conversation.” (Paediatrician 14)*
	**Social context of ADHD**: an understanding of the social context of ADHD informed the way some clinicians guided their patients at transition. Respondents also mentioned that some clinicians (and members of the public) understood very little about ADHD, or held outdated views.	*“And certainly we're still in a sort of stage with medical practitioners who query the validity of the approach. So GPs who won't prescribe, we're still in that sort of position locally.” (Paediatrician 18)* *“One of the Adult Mental Health consultants is very experienced and very good, but obviously that’s one out of probably ten. The others vary from no experience to moderate experience with one or two still on the ‘ADHD doesn’t exist within the adult population’ theory.” (Paediatrician 11)*
**As exchanged between services**	**Information exchange:** effective information sharing between child services and AMHS was seen as essential to support continuity of care: to inform AMHS about a new referral and child services about which referrals would be accepted. Information sharing practices varied from non-existent, to well established and effective. Sharing information with other agencies was rare, but could help to inform good transitional care.	*“…we have a transition panel...that meet every month, Adult and CAMHS representatives attend from all the teams and you bring [details of] anybody you want to transition.” (Child Psychiatrist 13)* *“For me it feels like they’re out there, you write to them but you don’t have any real liaison with them.” (Paediatrician 12)* *“…very joined up with work with CAMHS, with paediatricians, hopefully schools and education as well, primary care. So I think we do try to do things as the best we can but everybody has too much to do and not enough time.” (Paediatrician 14)*

^a^Participant job title and unique interview number; as detailed in the CATCh-uS report [21]

**Table 2:** Theme 2. Sharing information with young people

Sub-theme	Summary	Illustrative quotes (^**a**^)
**About the transition process**	**Timing**: the age at which clinicians started sharing information about transition with patients varied from starting at age 13, to just before age 18. Timing varied depending on multiple factors, such as available clinic time, the ‘readiness’ of the patient, and usual practice of the clinician. The majority reported starting around age 16/17, though some reported changing their practice recently to start talking about transition earlier.	*“…some of these families are just moving from crisis to crisis and the appointment is spent more trying to support them through the crisis or specific difficulty meaning that there’s less time to talk about transition or plan the transition. I think my personal practice at present is that it’s been quite late.” (Paediatrician 20)* *“It depends, you estimate what their trajectory is going to be partly depending on their functioning and all their other needs and their academic engagement, family engagement, their history, their responsibility so it depends on the young person’s needs.” (Child Psychiatrist 08)* *“So from a much younger age I’m now talking about this. … about 2 years ago so I was seeing cases that were 17 and a half where there had been no discussion about what was going to happen to them in adulthood. … So none of those conversations had been had so I had to very quickly go through that. But now I’m having those sorts of conversations from a very young age…” (Paediatrician 05)*
	**Content**: details provided to patients varied from none, to clear descriptions of the process. Where local transition programmes were in place, clinicians reported detailed discussions. Where clinicians felt uninformed, they were unable to provide patients with clear information.	*“So we've implemented Ready, Steady, Go here which is a transition programme from 13 onwards, kind of discussing transition with younger people from the age of 13 if we think they're going to be transitioning to adult services.” (Paediatrician 05)* *“I think sometimes we feel that we haven’t got much information to give a young person and parents because we don’t really… apart from knowing that there’s not much out there, we are not really fully informed about what other support they might be able to access.” (Paediatrician 19)*
	**Signposting**: several clinicians reported signposting young people on how to access adult healthcare, usually via their general practitioner (GP). This was described as a vague process, with an acknowledgement that they could be clearer.	*“...the advice is usually… because it could be anytime in their future, that their GP is their portal and knowledge of the services and that’s the best place to start.” (Child Psychiatrist 06)* *“I've certainly pointed them in the direction of the Adult Service already in the discussions so maybe I could be more explicit, I’m implicitly explaining that the family doctor would then be responsible…” (Paediatrician 18)*
	**Expected differences in AMHS**: informing young people and their parent/carers about differences between child and adult healthcare services when approaching transition was important. It could help manage anxiety, and prepare families for differences in information sharing practices. Several clinicians noted that young people might need more organisational support from parent/carers when in AMHS than previously in child services.	*“The biggest thing that young people have fed back to us is that they are just nervous about the differences. …so we kind of wanted them [AMHS] to write that out and sort of say you will see your doctor for medicine and this is when you will see your team worker and you will see them there or you see them there.” (Child Psychiatrist 13)* *“… we had a bit of a bad experience with one who was expecting the same service as they were getting from us….And they were quite irritated that they didn't and now I'm making it clear to patients ….They will only manage the ADHD. So I've made that much clearer now ... (Paediatrician 05)”* *“So it is an issue for the 18 to 25s I must say because they've suddenly got to go from the detailed support to very limited and dependent on parents much more.” (Paediatrician 12)*
**About self-management**	**Discussing the future**: some clinicians saw talking with patients about living with ADHD as a long-term condition as a key part of transitional care. These discussions helped to engage with the young person and helped them to think about self-management and possible future challenges. Others thought that they did not have the expertise to provide this kind of information, or said a specialist nurse or support group might be better.	*“I talk with them about their hopes and plans and aspirations and the need to take medication. (Paediatrician 17)”* *“So we would normally start that conversation and say ‘Right we’ve reached the conclusion that you meet the criteria for ADHD’ and then we’d say ‘And this is what it is’ and that would include long-term outcomes and prognosis and lifespan type discussions which we then just come back to every time we see them… So it’s ongoing.” (Child Psychiatrist 08)* *“We’ve tried to maintain contact and engagement over time and then in those youngsters there would be some that seem to be coping well and developing self-management strategies and we hope through running a teenage group to sort of help them understanding their own needs and self-management.” (Paediatrician 16)*
	**Healthcare into adulthood**: some clinicians encouraged young people to engage with decision-making about their care; a part of helping them learn to make informed choices. Some said that if teenagers questioned their treatment, it could be an opportunity to start to educate and engage them in self-management. A few emphasised approaching these conversations as a dialogue.	*“I don’t actually think that I have the level of expertise to really give an authoritative answer.” (Paediatrician 21)* *“The specialist nurse is fantastically good with younger adults, I feel a little bit lost.” (Paediatrician 15)* *“Try and help them understand that it often does help in college and that they don’t have to take it… they can take it on a flexible basis, … and so take things more into their own hands. So yes, we always have that conversation.” (Child Psychiatrist 01)* *And they start kicking off a little bit and saying 'I don't want to take it'. So then I use the opportunity to have a discussion about 'Okay why are you on this medication? Are you going to be on it for a long time? And if not what are the alternatives? …And I kind of give them my approach up to medication.” (Paediatrician 05)* *There’s no point in trying to persuade a young person to take medication against their wishes. ...it’s a dialogue, isn’t it? You have to have a kind of to and fro and involve young people. (Paediatrician 04)*
	**Self-management**: clinicians said that information exchange with young people about ADHD in adulthood was key to help them learn to self-manage; however, this was often difficult to do in the clinical time available.	*“And one of the opportunities about ADHD is you get the chance though for years to be able to chat to young people and give them more control and more power over it and that helps them to see it just as part of them, that they can get on with and manage. So I think that’s an important bit. It’s the transition within the young person’s development I think is very important.” (Child Psychiatrist 06)*
**To support engagement**	**Joint meetings**: including patients in joint meetings with child and adult mental health services was described as an effective way to make information about transition accessible to the young person, and ease anxieties. Joint meetings were only offered in a few cases.	*“…when I do handovers of other sort of patients to the Adult Psychiatrist I offer to do joint appointments and most of the kids seem to quite like that as part of the handover. So yes…”*
	**Information resources**: provision of information resources, such as leaflets, or website links, was variable, and often not ADHD specific. Some clinicians expressed frustration at the lack of quality information resources available, observing that mixed media and online information would be most acceptable for young people.	*“We have transitional leaflets and information leaflets for the families that I can give them anytime from 16…” (Child Psychiatrist 03)* *“…we haven’t got anything around ADHD specifically but we’ve got a couple of very good autism self-help resources in XXXX [local service].” (Paediatrician 11)* *“But no, we don’t have anything really good to hand… sort of written information about adult services. .... We don’t have a website, adults don’t have a website. Our young people actually would probably prefer even… they look a bit horrified when you hand them booklets these days. [Laughs] They all want to go online and google it and stuff like that.” (Child Psychiatrist 13)*
	**Allocated person**: the majority of clinicians identified that having an allocated person for contact and information could play a vital role in ensuring a good flow of information to the young person and their family through transition. This could be a specialist nurse, or support worker.	*“…allocated a social worker from adult services as they were coming up towards transition who then was really quite helpful in terms of supporting the young person, giving them advice about ...different things they could use to help themselves, organise themselves and so on and so forth.” (Paediatrician 09)*
**Accessible communications**	**Content and timing**: timing for discussing transition varied. Many clinicians tailored the content and timing of information they shared to make it more accessible, depending on the young person’s situation, and perceived readiness.	*“…it depends on the young person’s needs and normally we know them quite well by that age.” (Child Psychiatrist 08)* *“…so it depends when we think it’s the right time to have that conversation … I think it’s very much if an individual is not triggered necessarily by a particular age you take it as it comes.” (Paediatrician 16)*
	**Support with understanding**: young people with ADHD often need additional support in navigating information, including key information about transition. Meetings across services helped assisting with this.	*“With any luck, if you give them the information somewhere it will sink in and if things do go wrong they’ll come back.” (Paediatrician 11)* *“Ideally, the first appointment would be a joint handover...Because it’s impossible to get all the information on the forms and stuff like that, isn’t it, so I think sometimes just being in the room can help a little bit and you maybe remember stuff better than the young people.” (Child Psychiatrist 13)*
	**Including parents/carers**: parents and carers frequently play a key role in helping young people to navigate and take in healthcare information. This could cause difficulties at transition, especially as AMHS do not routinely include parents/carers in communications.	*“…they all nod and say 'uh huh' but it tends to be the parents who take on that information more.” (Child Psychiatrist 08)* *“Well usually both of them [parents and young people]. … They seem to be quite comfortable not to speak to us on our own.” (Paediatrician 15)*

^a^Participant job title and unique interview number; as detailed in the CATCh-uS report [21]

### Ethics

Ethical approval for this element of the study was granted by NRES South Yorkshire Ethics Committee: Yorkshire & The Humber (REC Reference: 15/YH/0426) and the University of Exeter Medical School Research Ethics Committee (REC Application Number: 15/07/070).

## Results

A total of 22 clinicians were interviewed from children’s services. These were 15 paediatricians and seven child and adolescent psychiatrists. They were located across 11 regions of the UK and within a variety of local adult ADHD service models. Only one region, the North West, was not represented. The sample is described in greater depth in the NIHR CATCh-uS report [21].

Two themes were identified that represent key data in relation to information provision as part of health service transitions for young people with ADHD:

1. Information for clinicians
2. Sharing information with patients

These themes are summarised below, and presented in Tables 1 and 2, with a summary of sub-themes, and illustrative quotes. For a full and detailed description of each theme, please see supplementary material. Theme one, *information for clinicians* is about the information that clinicians working in children’s services need in order to deliver practical elements of the transition process, and provide appropriate clinical advice, treatment, and care to young people with ADHD approaching the age of transition. Sub-themes include information about AMHS, information about the young person, and living with ADHD as a long-term condition. Also information exchange between services. Theme two, *sharing information with patients* is about the information clinicians reported sharing or wanting to share with young people to facilitate transition. This includes information about the transition process and what to expect. Other sub-themes included information about self-management into adulthood, relevant information to increase healthcare engagement, and tailoring information sharing methods to ensure young people with ADHD are able to access and understand key facts/concepts.

**Fig. 1:**
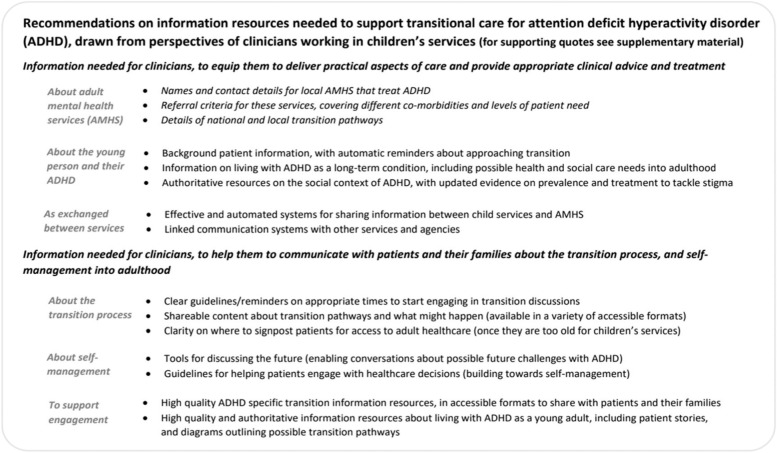
Recommendations for national provision of information to facilitate healthcare transitions for young people with ADHD

Recommendations on the types of information resources needed to support clinicians delivering transitional care were drawn from interview data. These include resources for direct use by clinicians, and for sharing with patients, and are presented in Fig. 1.

## Discussion

### Summary of findings

Clinicians in this study felt that accurate, tailored and easily available information is a critical element in the transition process for young people with ADHD, as reflected in the two themes presented in this paper: Information for clinicians themselves, and information shared with young people. Across these themes, clinicians discussed points of best practice around the accessibility of information and information sharing as a longer-term process.

Clinicians required up-to-date information and resources to share with young people, as well as understanding and awareness of the social context for their patient. If available, this allowed them to engage in a backwards-and-forwards flow of information with the young person. This flow of information was seen as facilitating self-management during transition, for example allowing young people to try out making decisions around medication and support and reviewing what happens afterwards.. Where information was not available, easily accessible or out-of-date, however, this was viewed as having consequences both for the process of transition between services, and for the young person’s progress with understanding and managing ADHD into adulthood. We highlight some of the most important information gaps to fill in terms of policy, practice and research. These should also be viewed in the light of existing literature on other identified barriers and facilitators to transition for young people with ADHD, which are often interwoven and are likely to require integrated solutions [21, 27, 34, 45]. Identified barriers include cultural differences between child and adult services [5, 34], lack of shared transition planning between services [37], the unsupported ‘default’ role of primary care in treatment of adult ADHD [28], premature discharge from services [34], unmet need for accessible and age-appropriate services [33], the need to tackle stigma [11], variable attitudes towards medication [21, 50], and lack of training in and awareness of adult ADHD [18, 25].

### Information for clinicians

Guidance often seems to take for granted that the clinician has the appropriate information to start with, and that clinicians then ‘impart’ the information to the young person or use it to negotiate and arrange care. For many clinicians in these interviews, this was clearly not the case, either for information about transition processes, or about ADHD as a long-term condition. Whilst some clinicians were aware of local transition protocols, for others, the lack of such protocols (or their lack of accessibility) made it much harder to negotiate the pathways and processes. Previous research suggested that many clinicians were not aware of their Trust’s transition protocols [19]. Similarly, participants in our interviews often lacked information about the availability and the acceptance criteria of local AMHS, which supports the findings of the CATCh-uS mapping study that many respondents were not aware of existing (dedicated) ADHD services [33]. Some clinicians perceived there to be a lack of information available to them about ADHD as a long-term condition. This may be linked to the fact that ADHD itself has been a frequently contested diagnosis, particularly in adults, while health professionals still indicate a lack of confidence in management of, and even in the validity of, the diagnosis [20, 28, 46].

### Information for young people

In these interviews the clinician’s own uncertainties or gaps in up-to-date knowledge often translated into how and to what extent they shared, or were able to share, information with young people. Clinicians felt that the information they delivered and discussed with young people, as well as enabling access to AMHS, had a particularly important role in decisions around medication at the time of transition and in allowing young people to ‘test’ stopping treatment. Whilst many young people may no longer meet criteria for ADHD in adulthood, symptoms continue into adulthood for up to two thirds of children and adolescents, and a minority continue to meet full diagnostic criteria [2, 4]. As a result, as young people approach the age for transition there is likely to be a need for assessment and decision-making about any individual’s need for longer-term care and support. For a clinician working in children’s services, it will be necessary to use clinical judgement and consultation with the young person and their family to explore and test over time whether to reduce and stop treatment before adulthood, or whether ongoing treatment may be needed. These discussions and joint decisions need to be informed by a nuanced understanding of ADHD as a potential lifelong condition, and awareness of how presenting symptoms and behavioural challenges may develop and change as the young person approaches adulthood.

Therefore, conversations around treatment during transition were seen not just as a factual transfer of information but as a communication of possibility, risks and benefits, informed by dialogue with the young person, the clinician’s experience and prior knowledge, and their understanding of individual needs and family context. It was also informed by the clinician’s own views of the role of medication in ADHD. In some interviews, clinicians expressed uncertainties around the benefits of medication even where young people continued to have symptoms. However, it is also worth noting that a prominent theme in our parallel interviews with young people who had dropped out of CAMHS and re-entered services as adults, was that in retrospect they wished clinicians had emphasised the benefits of remaining on medication if warranted by symptoms and impairment rather than presenting the decision as a neutral choice [21].

Another important aspect was the sharing of information with young people about ‘what to expect’. The differences between child and adult mental health services, young people’s expectations of AMHS, and how young people actually experience AMHS has been a strong theme in prior research with clinicians and with young people [3, 5, 45]. Differences in parental involvement are often highlighted in our interviews; clinicians commented that transition to AMHS could, in fact, require greater parental involvement than care under child health services, owing to the need to organise and manage the volume and flow of information involved in transfer of care and the less ‘holistic’ approach of AMHS. When this is added to the difficulties inherent in ADHD with attention and information processing [49], it is clear that young people without an involved parent are likely to be at a significant disadvantage during the process of transition. This can further widen inequalities. Findings from our work with young people and parents underlines the importance of having a parent/carer or other supporter to seek, navigate and translate information about ADHD into adulthood, and transition between services, to facilitate engagement with services [21, 35].

#### Sharing information

Clinicians reported signposting young people onto other services and sources of information and advice, as well as advising young people on how they can get back in touch with services if they need to re-enter. Interestingly, this contrasts with accounts from qualitative research with young people, where many report a lack of information in terms of not knowing where to go, and not being aware of how they can re-enter services [21, 35]. These differing accounts may partially relate to the form and presentation of information shared; and what young people are able to understand and retain during healthcare appointments. Clinicians themselves voiced a need for evidence-based, good quality and consistent information that they can provide, and signpost young people to, in formats that are accessible; instead of using material not designed for ADHD or taken from other services. It is also worthwhile noting that some clinicians in this study reported advising young people that their GP would be able to provide advice and act as a portal to services. Whilst this may well be true in these individual cases, research with GPs suggests that primary care professionals often feel ill-equipped to take on such a role; and also needed better information about the condition and the support available [16, 28]. It is crucial that those whom young people approach for information are as well-informed as possible, as misinformation (however well-intentioned) can cause later distress for young people and families [35].

#### Strengths and limitations

This paper presents an analysis of a substantial number of interviews with clinicians from child services, across a range of different NHS Trusts and geographical areas. The sample was recruited as part of the CATCh-uS study, which had key research questions and topic guides built around the process of transition, with no specific focus on the role of information. Further themes may have been identified had we systematically enquired about the role of information. In our analysis however, it became clear that information was a significant topic with important implications for practice, meriting separate presentation. Future research could explore this issue more systematically.

#### Implications for practice and research

Our findings clearly suggest the need for efficient and simple ways of sharing information about transition in ADHD with clinicians. Adult ADHD remains one of the more contested conditions, affected by stigma and by under-provision of services [33], and this arguably warrants additional efforts being made to ensure that information is as easily available as possible. Information technology offers cost-effective methods of providing nationally available high-quality evidence-based information at the point of need, to support existing mechanisms of healthcare transition. Information on ADHD as a condition, tailored for each stakeholder group (clinicians in child and adult services, general practitioners, young people, and parent carers) may reduce the impact of some barriers to continuity of care. For example, nuanced information on how an adolescent with ADHD may continue to need treatment and support as an adult, may help to tackle stigma, reduce premature discharge from services, inform attitudes towards medication, and enhance awareness of adult ADHD. Routine and systematic provision of information about care pathways and locally available services to clinicians working in children’s services would make it easier to prepare young people for what to expect in adult services, and help to highlight gaps in care, stimulating work to find solutions to enable continuity of care for the young person. There are likely to be roles for a number of different agencies to improve availability and quality of such information, such as NICE, ADHD charities, and NHS informatics. With the increasing use of online or digital systems within services, there is clearly scope for linking to central or regional portals or patient information management systems through which clinicians can access up-to-date information about the condition, and about available services. These systems could also use temporal flags with prompts for topics to be discussed with patients well in advance of the age boundary, in accordance with NICE guidelines.

Given the importance of decisions made at this life stage, it is crucial that clinicians have access to up-to-date evidence on the benefits and harms of medication to enable them to have discussions with young people about their treatment. There is much more to be known about outcomes for young people with ADHD and the optimal approach to management of the condition into adulthood and beyond, such as the longer-term effects of medication [22].

There may be a place for tools for clinicians to support young people’s decision-making around transfer to AMHS and continuation of medication. Such patient decision aids have been developed for parents of children with ADHD [9], and for young people with other chronic conditions such as diabetes, e.g. Lawson et al. [23], but to our knowledge these aids have not been evaluated for young people with ADHD, representing an important avenue for co-creation and research. Although many clinicians will know their patients well, those in transition may sometimes be overlooked; therefore systems which can flag those who might be less well supported and smooth the flow of information could also be valuable. Our parallel paper [35] provides recommendations for accessible, co-created and trusted resources for young people. Research is also required to explore the most effective ways to share information that young people with ADHD can understand, retain, and use for self-management and to seek support if required. This may involve trialling information-based interventions and exploring their effectiveness in improving the outcomes of transition. NICE guidelines now emphasise the importance of the provision of information in an accessible format, as part of care for people with ADHD [30]. To facilitate such provision, NICE could produce or host information resources as part of their Tools and Resources website.

## Conclusion

In conclusion, information plays an important role for clinicians caring for young people with ADHD approaching transition, enabling them to support, provide appropriate care, and inform the young person. This is perceived as having a clear link to smooth transition and improved outcomes for young people. Information flow and quality is too important to be left to chance and resource-poor local services. While clinical guidelines on transition and management of ADHD [29, 30] have been updated to highlight the importance of information provision, there remains much to be done to support clinicians in implementing these guidelines, and to avoid local variations in care where transition may depend to some extent on local expertise and knowledge. Our research adds to the case for investment in research, development, and nationally available materials for clinicians and patients.

## Supplementary Information

**Additional file 1.** Themes and sub-themes in details, with illustrative quotes.

## Data Availability

The datasets generated and/or analysed during the current study are not publicly available but are available from the corresponding author on reasonable request. Data is currently stored securely by the University of Exeter College of Medicine and Health.

## Abbreviations

ADHD: Attention Deficit Hyperactivity Disorder
AMHS: Adult Mental Health Services
CATCh-uS: ‘Children and Adolescents with ADHD in Transition between Children’s and Adult Services’ study
CAMHS: Child and Adolescent Mental Health Services
GPs: General Practitioners
NHS: National Health Service
NICE: National Institute for Health and Care Excellence
NIHR: National Institute for Health Research
NRES: National Research Ethics Service (now the Health Research Authority)
UK: United Kingdom
