# Supplementary material for: In transition with attention deficit hyperactivity disorder (ADHD): children’s services clinicians’ perspectives on the role of information in healthcare transitions for young people with ADHD
Source: BMC Psychiatry. 2022 Apr 9;22:251. doi: 10.1186/s12888-022-03813-6 (PMC8994234; doi:10.1186/s12888-022-03813-6)
Supplement: Supplementary file 1 — Additional file 1. Themes and sub-themes in details, with illustrative quotes. [file 12888_2022_3813_MOESM1_ESM.docx]

# Supplementary material

Themes and sub-themes in details, with illustrative quotes.

# Theme 1. Information for clinicians

This theme is about information that clinicians working in children’s services need to deliver practical elements of the transition process, and appropriate clinical advice, treatment, and care, while also facilitating continuity of care into adulthood for young people with ADHD approaching the age of transition.

## About Adult Mental Health Services (AMHS)

### Which service:

The majority of clinicians interviewed reported limited or non-existent information about where and how to refer young people with ADHD to once too old for their service.

*I have no idea what their criteria are or whether they have changed...I don’t know the rationale behind that. (Paediatrician 02*)*

*There’s supposed to be a kind of transition path that we could give to young people...we haven’t managed to get that yet and we kind of feel that we can’t move forward with it. (Child Psychiatrist 13)*

The few clinicians reporting local transition pathways, which included names and contacts for local AMHS, said that this made supporting transition straightforward.

*We now have a psychiatrist who, part of the time, specialises in adult ADHD, so we now refer to her. So that makes life a lot easier and means that it’s much more straightforward. (Child Psychiatrist 01)*

### Referral criteria:

For many clinicians, unclear AMHS acceptance criteria made gathering accurate referral information a complex and difficult process. Clinicians reported that AMHS differed in accepting a referred patient, depending on locality, level of complexity, medication status, and existing co-morbidities. Clinicians also reported cases where no AMHS would accept a referral, or where information about which referrals they would accept was difficult to access.

*If they have got autism and ADHD and they aren’t on medication it can get really messy... Because there’s no pathway. (Paediatrician 11)*

In some areas, clinicians reported that while they were able to transfer patients with co-morbidities, they were unable to find an AMHS that would accept a patient with ‘straightforward ADHD’, despite ongoing health care needs.

*If there are additional mental health issues, so a child, for example, who suffers with a lot of anxiety issues, then we can generally refer them on to Adult Services but not if it’s ADHD alone. (Paediatrician 19)*

Young people not currently taking ADHD medication were not likely to be referred.

### Transition pathways:

Many clinicians were not familiar with the existence of the NICE guidelines on transition, and/or described them as not being specific enough.

*I generally find them [NICE guidelines] useful. I can’t really recall whether they have anything particularly to say about the transition.* (*Paediatrician 02)*

Where available, local protocols/guidelines were seen as providing helpful and concrete transition information. However the majority reported a lack of clear pathways for ADHD transition, which undermined transitional support for young people.

*When I first started we didn’t have that and it was a bit more, you know, kind of phoning and looking for people and saying, ‘Are they this team or that?’ So the protocol has massively improved our transition. (Child Psychiatrist 13)*

### Transition outcomes:

The majority of clinicians reported having no information about what happened to young people after leaving their service, or whether they transitioned successfully. Some expressed unease at this.

*I would assume many [drop out at transition] but I don't have any knowledge of any. We don't have any effective data monitoring system to know what happens. (Paediatrician 18)*

A few clinicians reported gathering information, to check whether or not young people had attended appointments with Adult Services.

*I will often give them that extra follow-up to make sure that they've come to rest safely. (Paediatrician 18)*

*I know from the Adult Services that some of our people we do refer just don’t attend. (Child Psychiatrist 01)*

## About the young person, and their ADHD

### About the young person:

Several clinicians described methods of gathering background information about their patient to inform transitional care decisions, with specialist nurses often fulfilling this role. This included gathering contextual information from home visits, conversations with parents, and gaining updated information from the young person’s school, as needed.

*…keep some sort of intermittent dialogue perhaps through a telephone clinic with the parent just to check all is well, and if all is well then we would discharge them, but if they were still concerned we would tend to get updated information from school in the form of questionnaires …if they look awful then make contact with the parent again and then invite the child to have another chat about life, and if it looks good then we would discharge at that point. (Paediatrician 16)*

Some clinicians said that an IT system to automatically flag up and remind them when patients are approaching transition would be useful.

*We could really do with something that flags up a reminder to clinicians to start thinking at 16 about transition or earlier if need be and we don’t have that…* *Our systems are very primitive. (Child Psychiatrist 03)*

One clinician described how when they have very little background information about a patient, often as a result of the structure of their service, it makes it difficult for them to support the transition process for a patient in an informed way.

*Sometimes I’ve only seen them once and then I’m discussing transfer and I’m thinking I hardly know… I don’t have a lot to add to any sort of transfer information. …so I think that’s very frustrating and probably feels rather poor. (Child Psychiatrist 07)*

### About ADHD as a long-term condition:

Clinicians found that providing information about ADHD as a long-term condition could be a challenging task, due to clinical uncertainty over how symptoms might evolve in an individual young person and affect their need for medication and other treatment. One clinician emphasised their role in providing an informed clinical perspective about ADHD, which was contrasted with other long-term conditions such as diabetes, where the need for treatment into adulthood is more certain. Depending on the information they held, and their professional understanding, clinicians conceptualised and explained possible outcomes differently.

*I’d say a majority still need it. Some do seem to outgrow their ADHD and that’s great, and I have had that happen, but I’d say a majority still need ongoing management with medication into adulthood. (Paediatrician 09)*

*…there is also a sense that as young people grow older, that they need it less because they are able to self-manage and understand their strengths and needs... (Paediatrician 02)*

Some clinicians appeared well informed, and spoke in detail about likely changes young people with ADHD might experience.

*…with ADHD, normally the hyperactivity has settled down by then, the impulsivity is there to a certain extent, it's the disorganisation that's a big problem and the emotional dysregulation… (Paediatrician 05)*

Others highlighted a perceived lack of up-to-date and accurate information on ADHD as a long-term condition, and on likely outcomes for the young person. This could make facilitating transition difficult. It was suggested that creating transition services could offer a solution, as they would have more up-to-date and relevant information for young people.

*So if there was a transition service … to link in with that process that would work really nicely in terms of … advising things like driving and other careers and careers that they are excluded from if they are on medication. Sometimes we probably don’t have all the up-to-date information, or have to go away and research it ourselves to make sure that we are not giving the wrong advice. (Paediatrician 19)*

### Social context of ADHD:

An understanding of the social context of ADHD, including how GPs and Adult Service teams are likely to conceptualise ADHD, may help the clinician to guide young people effectively through the transition process, or at least to understand potential barriers to ongoing care and support. One clinician explained how this informed their discussions with young people and their families.

*…there are so many different issues. I suppose what I'm trying to say is that for something like ADHD where the diagnosis has a whole lot of subjective socially bits coming into it …* *So I think that I can give an experienced medical take on that in something where there isn't a right or a wrong answer. We can have this conversation (Paediatrician 14)*

Respondents mentioned that some clinicians (as well as the public) understood very little about ADHD, as a result of outdated information and theories about the condition, with some even doubting the validity of ADHD diagnoses in adults. They also talked in terms of social understanding of the condition changing and improving over time.

*And certainly we're still in a sort of stage with medical practitioners who query the validity of the approach. So GPs who won't prescribe, we're still in that sort of position locally. (Paediatrician 18)*

*One of the Adult Mental Health consultants is very experienced and very good, but obviously that’s one out of probably ten. The others vary from no experience to moderate experience with one or two still on the ‘ADHD doesn’t exist within the adult population’ theory. (Paediatrician 11)*

## As exchanged between services

### Information exchange:

Effective information sharing between services was seen as a central part of a successful and supported transition, and necessary to facilitate continuity of care into adulthood. The functions of information sharing included allowing the Adult Service to prepare to meet the needs of the young person moving into their care, and informing the Child Service clinician about which referrals would be accepted. Clinicians reported information sharing practices that varied from non-existent, to well established and effective. Some described routine information sharing between services, including transition meetings; others described a perception that communication with Adult Services was only one way.

*…we have a transition panel...that meet every month, Adult and CAMHS representatives attend from all the teams and you bring [details of] anybody you want to transition. (Child Psychiatrist 13)*

*For me it feels like they're out there, you write to them but you don't have any real liaison with them. (Paediatrician 12)*

Information sharing with other agencies, such as education or social care, about transition, was helpful both to inform clinical care, and support the young person to navigate other life changes such as changing education setting. However due to time and resource constraints, in practice this form of information exchange was often ‘ad hoc’, rather than forming part of an established process.

*…very joined up with work with CAMHS, with paediatricians, hopefully schools and education as well, primary care. So I think we do try to do things as the best we can but everybody has too much to do and not enough time. (Paediatrician 14)*

*…if I know that the family has got a social worker, again, I would copy them in to keep them in the loop of what the plan was. We do try and ask to be invited if there are any meetings, child in need meetings and so on but, I don’t know, it’s a bit variable. Sometimes I get to go to them and sometimes I don’t know anything about them. (Paediatrician 09)*

# Theme 2. Sharing information with young people

This theme is about the information clinicians reported sharing, or wanting to share, with young people, to facilitate transition. This includes information about; the transition process, and what to expect; ADHD as a long-term condition, and self-management into adulthood; relevant information to increase healthcare engagement; and tailoring information sharing methods to ensure young people with ADHD are able to access and understand key facts/concepts.

## About the transition process

### Timing:

The timing of conversations to communicate information about transition appeared to depend on a range of factors including; local protocols and practical issues such as available clinical time, the perceived needs and readiness of the young person to take in this information, and individual practice of the clinician. Although variable, the majority reported starting to share this information with young people from ages 16 to 17 years.

*It depends, you estimate what their trajectory is going to be partly depending on their functioning and all their other needs and their academic engagement, family engagement, their history, their responsibility so it depends on the young person's needs. (Child Psychiatrist 08)*

*…some of these families are just moving from crisis to crisis and the appointment is spent more trying to support them through the crisis or specific difficulty meaning that there's less time to talk about transition or plan the transition. I think my personal practice at present is that it's been quite late. (Paediatrician 20)*

Some clinicians reported they had begun to share transition information with young people at an earlier stage, avoiding the rush to address this just before their 18^th^ birthday, and providing time to have conversations about different aspects of the process.

*So from a much younger age I'm now talking about this. … about two years ago so I was seeing cases that were 17 and a half where there had been no discussion about what was going to happen to them in adulthood. Who was going to prescribe their meds, what they were going to say to their employer, what impact this had on anxiety and depression throughout life and other things. So none of those conversations had been had so I had to very quickly go through that. But now I'm having those sorts of conversations from a very young age… (Paediatrician 05)*

### Content:

In cases where clinicians knew of an adult service that would accept the young person, and especially where their transition practice was supported by a protocol or programme, they reported sharing detailed and nuanced information over time.

*So we've implemented Ready, Steady, Go here which is a transition programme from 13 onwards, kind of discussing transition with younger people from the age of 13 if we think they're going to be transitioning to Adult Services. (Paediatrician 05)*

Others, who were not aware of an appropriate Adult Service, told the young person their care would be handed over to the family GP, or that they would find out where the young person could go. Some clinicians reported sharing uncertainty with the young person over what might happen next. This included the lack of an identified provider for medication support, or explaining to young people who were not on medication that there was no Adult Service for them.

*… I would start talking to them then …about who the right person to monitor you is…And I normally say 'And I don't expect that will be your GP' because I don't know one day a GP might say 'Yes' but I don't think they will do locally. And then I normally say to the child and young person and to their family 'And I'll find out who that is'. Because I sort of do think that's my job to do that. (Paediatrician 14)*

*I just talk them through it and say, ‘Look, I can’t refer you on to a resource afterwards because you are not on medication, so there’s nothing I can actually refer you on to’. (Paediatrician 11)*

Some clinicians observed that their ability to provide clear information to the young person depended on how much they knew themselves about available options.

*I think sometimes we feel that we haven’t got much information to give a young person and parents because we don’t really… apart from knowing that there’s not much out there, we are not really fully informed about what other support they might be able to access. (Paediatrician 19)*

### Signposting:

Clinicians discussed strategies for signposting young people so that they could access services when needed, and know where to go for advice and support. Clinicians often reported advising young people that their GP could act as their portal to services.

*...the advice is usually… because it could be anytime in their future, that their GP is their portal and knowledge of the services and that’s the best place to start. (Child Psychiatrist 06)*

Descriptions of the signposting process varied from a vague communication of information, which seemed to place responsibility for gaining appropriate information onto the young person, to an acknowledgement that they could be more explicit in sharing this information.

*So they're given advice that they should seek support but essentially they're adults with capacity who need to work their own way out as to who the most appropriate person is but they'll get given advice and suggestions when they're discharged. (Child Psychiatrist 08)*

*I've certainly pointed them in the direction of the Adult Service already in the discussions so maybe I could be more explicit, I’m implicitly explaining that the family doctor would then be responsible… (Paediatrician 18)*

Some clinicians reported difficulties associated with sharing information with young people with ADHD, with one observing that in practice it was often parents who took the information in. See Theme 2D.

### Expected differences in AMHS:

Important information about differences between Child and Adult services included the likely move away from family-based and holistic support, to a more individual focus on the young person and their ADHD. Clinicians said that communicating about these differences in advance could help young people and their families to anticipate and better manage the coming changes. Several said that they had learned through experience that they needed to provide this information more clearly, as the lack of it could increase anxiety for young people.

*… we had a bit of a bad experience with one who was expecting the same service as they were getting from us….And they were quite irritated that they didn't and now I'm making it clear to patients ….They will only manage the ADHD. So I've made that much clearer now ... (Paediatrician 05)*

*the biggest thing that young people have fed back to us is that they are just nervous about the differences, what’s going to be the differences between adult and young people, and I guess one of the big differences is about that’s slightly more separate. …so we kind of wanted them [Adult Services] to write that out and sort of say you will see your doctor for medicine and this is when you will see your team worker and you will see them there or you see them there. (Child Psychiatrist 13)*

Clinicians also described preparing patients and parents/carers for differences in information sharing between Child and Adult Services, and the need for parents to support young people with organisation. It was recognised that this support might be required to a greater extent than for neuro-typical young people, and that those without support might particularly struggle as Adult Services would not routinely contact parents directly.

*I have had those conversations with patients, preparing them for the fact that at 18 they get invitations to appointments, their parents are generally written to, … because often these kids with ADHD…actually still need quite a lot of guidance and help with their organisation by their parents so that their parents are more actively involved than routinely. (Child Psychiatrist 07)*

*We tend to do that* [keep contact] *via parents or carers. Whereas in Adult Services that isn't tolerated. So that subpopulation, they're the ones that really struggle. (Child Psychiatrist 08)*

Clinicians highlighted that leaving Child Services could lead to a greater dependence on parents for information and coordination, and that young people needed to be prepared for this.

*So it is an issue for the 18 to 25s I must say because they've suddenly got to go from the detailed support to very limited and dependent on parents much more. (Paediatrician 12)*

Theme 2D highlights the important role of parent/carers in helping young people take on information relevant to their healthcare transition.

## About self-management

### Discussing the future:

A few clinicians described communicating with the young person about ADHD as a long-term condition. They reported conversations about their diagnosis, the possible trajectory of their ADHD, and the impact that this might have on their life in adulthood, their relationships, work and social lives. Sharing information about what ADHD might be like into adulthood performed a dual function of informing young people about what to expect, and preparing them for the fact that they might need to transition, and engage with taking medication long-term.

*I talk with them about their hopes and plans and aspirations and the need to take medication. (Paediatrician 17)*

Those clinicians, who proactively shared this kind of information, saw it as encouraging young people to adopt a more engaged and independent approach to self-management. Some revisited the discussion at each appointment.

*So we would normally start that conversation and say 'Right we've reached the conclusion that you meet the criteria for ADHD' and then we'd say 'And this is what it is' and that would include long-term outcomes and prognosis and lifespan type discussions which we then just come back to every time we see them… So it's ongoing. (Child Psychiatrist 08)*

Others described these discussions on life and self-management information as taking place when appropriate, rather than on a routine basis.

*We would talk about sex and drugs and stuff when we think the time is appropriate, or if there were issues of concern around compliance with medication or having questions about medication and having difficulties then, and I think that would be a trigger for having a sort of one-to-one, quite a long clinical appointment to discuss life and stuff at that stage. So I think it’s very much if an individual is [ready], not triggered necessarily by a particular age you take it as it comes. (Paediatrician 16)*

Several reported providing whatever advice they could to help them manage their lives while transitioning into adulthood, while acknowledging that they lacked the expertise to be able to provide enough information and support.

*I don’t actually think that I have the level of expertise to really give an authoritative answer. (Paediatrician 21)*

Holding a discussion about healthcare options and self-management strategies, was seen by some clinicians as an important way of engaging the young person. Although one clinician felt skilled enough to have these discussions, others mentioned that a nurse specialist or specialist group might be better.

*I think I’m relatively well placed to tell them what the options are and I suppose that would be how I would try to do most things, it would be these are the options and I’ll give you some information I know about them and what do you think. (Child Psychiatrist 10)*

*The specialist nurse is fantastically good with younger adults, I feel a little bit lost. (Paediatrician 15)*

*We’ve tried to maintain contact and engagement over time and then in those youngsters there would be some that seem to be coping well and developing self-management strategies and we hope through running a teenage group to sort of help them understanding their own needs and self-management.* (Paediatrician 16)

### Healthcare into adulthood:

Several clinicians described discussing with patients the need for medication, and other support into adulthood. Approaches varied from an apparent discouragement of ongoing medication, to encouraging young people to trial periods without medication, to preparing the young person for the possibility that they might benefit from support into adulthood, depending on their life plans.

*I would start talking about medication or continue with it, not continue with it, probably around Year 11 [age 15/16], ..., I might say 'See how things go, have a break for a couple of weeks over summer holidays, go back onto it if you feel that it's appropriate or that it's helpful that if you don't want to carry on with it then come back and we can talk about you not being on it and what that might mean'. (Paediatrician 14)*

*Often they are at college. Try and help them understand that it often does help in college and that they don’t have to take it… they can take it on a flexible basis, they can take it on college days and not other days and so on and so forth and so take things more into their own hands. So yes, we always have that conversation. (Child Psychiatrist 01)*

Clinicians described dealing with complexity, where information sharing could help the young person to understand and test the nuanced picture of how, and to what degree medication might continue to help them. One clinician contrasted this with giving young people with diabetes information about insulin, which they saw as being much more clear-cut.

*So I think that I can give an experienced medical take on that in something where there isn't a right or a wrong answer. We can have this conversation, it's not like me saying 'You must carry on with this insulin for the rest of your life. You might benefit from this now, but you might not we don't know'. (Paediatrician 14)*

Some clinicians reported that teenagers may start to question the need for their ADHD medication, and described using this as an opportunity to start an education and engagement process with the young person.

*And they start kicking off a little bit and saying 'I don't want to take it'. So then I use the opportunity to have a discussion about 'Okay why are you on this medication? Are you going to be on it for a long time? And if not what are the alternatives? What are we going to do instead?' And I kind of give them my approach up to medication. (Paediatrician 05)*

Others laid out costs and benefits of medication into adulthood.

*‘Medication is not necessary. If you want to stop it, that’s something that I would support you in, but you need to know that it’s going to be a real struggle to keep yourself focused and out of trouble for the next three years and are you able to do that?’ Yes, sometimes it’s a matter of saying, ‘Okay…’ You give them a bit of leeway and you say, ‘Okay, have a go.’ (Paediatrician 04)*

A few clinicians emphasised the importance of approaching this information exchange as a dialogue, and listening to the young person as well as sharing their experience. One clinician referred to the two-way exchange of information, observing that it was important to listen to the young person during this process.

*There’s no point in trying to persuade a young person to take medication against their wishes, and sometimes a period not taking medication is a useful thing for everybody to demonstrate what the effects are and it gives a young person an opportunity to actually see what the benefits and the disadvantages...it’s a dialogue, isn’t it? You have to have a kind of to and fro and involve young people. (Paediatrician 04)*

Some clinicians reflected that a young person’s understanding of ADHD, and interpretation of their own healthcare needs were likely to impact on whether they continued to engage with treatment, and ultimately transition into Adult Services.

*…the kids who buy in to a model of a biological dimension to attention which is adversely affecting their skill experience which can be medically modulated, who have had a successful trial, who sort of get that model, generally stay with it. And the disaffected young people for whom this medicalisation is just yet another example of the system trying to screw them over, don't buy into it and default at the earliest opportunity*. (Paediatrician 18)

For more about the different ways this kind of nuanced information can be shared with young people, see Theme 2D.

### Self-management:

For some clinicians, exchange of information was key to enabling young people to make their own informed, decisions about their care, and ultimately their transition.

*And one of the opportunities about ADHD is you get the chance though for years to be able to chat to young people and give them more control and more power over it and that helps them to see it just as part of them, that they can get on with and manage. So I think that’s an important bit. It’s the transition within the young person’s development I think is very important. (Child Psychiatrist 06)*

Several clinicians talked about the limited time they had with patients. Where possible, discussions were had about medication management, and clinical care, but wider life issues, which interact with the need for medication, were difficult to cover in clinical time.

*…there are a lot of worries that young people have about staying on medication and also things like self-medication with other substances and interactions and all that sort of advice as well that we probably don’t do as well as we should be doing. (Paediatrician 19)*

## To support engagement

### Joint meetings:

Several clinicians mentioned that conducting a joint appointment between themselves, the young person, and a representative from Adult Services, was a good way of helping the young person to take in concrete information, and ease their anxieties about the next step. This was seen as leading to a smoother transition, and better attendance rates. Joint meetings were offered by some services, but not all.

*…when I do handovers of other sort of patients to the Adult Psychiatrist I offer to do joint appointments and most of the kids seem to quite like that as part of the handover. So yes, and if that was part of the protocol, then yes, that would be a good thing. (Child Psychiatrist 07)*

### Information resources:

When clinicians talked about resources they used to share information with young people to supplement conversations in clinic, these were usually written, and occasionally included links to online resources.

*We have transitional leaflets and information leaflets for the families that I can give them anytime from 16…. (Child Psychiatrist 03)*

Reports differed from service to service, and many reflected a pragmatic use of available materials, rather than a comprehensive resource, specifically designed for young people with ADHD.

*…we haven’t got anything around ADHD specifically but we’ve got a couple of very good autism self-help resources in XXXX [local service]. (Paediatrician 11)*

*…we start with ...the Ready, Steady, Go programme... it's on the XXX [local] NHS website, if you just Google Ready, Steady, Go, you can just download the forms for free and we're using that basically.* *(Paediatrician 05)*

Several expressed frustration at the lack of good clear information available to them for sharing with young people, especially in formats that young people could engage with. This was seen as both being due to limitations in their own knowledge, and because of a shortage of written materials, or accompanying online resources.

*…I think sometimes we feel that we haven’t got much information to give a young person and parents because we don’t really… apart from knowing that there’s not much out there, we are not really fully informed about what other support they might be able to access. (Paediatrician 19)*

*But no, we don’t have anything really good to hand… sort of written information about Adult Services. .... We don’t have a website, adults don’t have a website. Our young people actually would probably prefer even… they look a bit horrified when you hand them booklets these days. [Laughs] They all want to go online and google it and stuff like that. (Child Psychiatrist 13)*

Some identified a need for better quality online, written and visual information to engage young people with ADHD through transition, and share appropriate information in accessible ways.

*All we want is a page with pictures… they generally do want to just look at a picture and go, ‘Okay, that’s Dr So-and-so, that’s who I am going to see for my ADHD meds.’ It’s funny, it just makes them feel much more in control or something. (Child Psychiatrist 13)*

### Allocated person:

The majority of clinicians identified that having an allocated person to communicate information with the family from pre-transition through to engagement with AMHS, would have multiple benefits. This transition worker could be a specialist nurse, care-coordinator, or other health worker, who maintained contact and ensured a good flow of relevant information throughout transition, for the young person and their family. These workers could also help to inform the young person about ADHD as a long-term condition, and share strategies and advice about ADHD in adulthood.

*…allocated a social worker from Adult Services as they were coming up towards transition who then was really quite helpful in terms of supporting the young person, giving them advice about ...different things they could use to help themselves, organise themselves and so on and so forth. (Paediatrician 09)*

## Accessible communication

### Content and timing:

As discussed in Theme 2A, some clinicians appeared to make individual assessments about when and how much information to share with the young person based on circumstances. These assessments took account of the estimated life trajectory of that young person, their family context, plans, perceived risks and level of need, and how well the clinician knew that young person.

*…depending on what kind of education they go into post 16, and if they stay on medication, then I talk about what their transition would be* *from 17 years. (Paediatrician 17)*

*…it depends on the young person's needs and normally we know them quite well by that age. (Child Psychiatrist 08)*

Several clinicians emphasised possible risk levels, and readiness and ability to take in the information.

*…so it depends when we think it’s the right time to have that conversation and if there are sort of obvious adolescent issues and issues around risk taking or issues around sort of safety in driving, or we are aware of drugs and substances, then we would do that before...that would be a trigger for having a sort of one-to-one, quite a long clinical appointment to discuss life and stuff at that stage. So I think it’s very much if an individual is not triggered necessarily by a particular age you take it as it comes. (Paediatrician 16)*

### Support with understanding:

Several clinicians commented that young people with ADHD might struggle to take in information. Ways of supporting young people to take in information varied from hoping something would ‘sink in’, to aiming to provide good quality written information, so that young people could absorb information in their own time, as discussed in Theme 3B.

*With any luck, if you give them the information somewhere it will sink in and if things do go wrong they’ll come back. (Paediatrician 11)*

One clinician stated that a reason for the importance of joint hand-over appointments was because healthcare workers were more likely to remember key information than the young person.

*Ideally, the first appointment would be a joint handover...Because it’s impossible to get all the information on the forms and stuff like that, isn’t it, so I think sometimes just being in the room can help a little bit and you maybe remember stuff better than the young people. (Child Psychiatrist 13)*

### Including parents and carers in the exchange of information

Most clinicians described parents/carers as being included in the process of sharing information in Child Services. This often happened as a matter of course, because the young person would visit the clinic with a family member.

*Well usually both of them [parents and young people]. … They seem to be quite comfortable not to speak to us on our own. I think very few, I would say a handful of them after 16 have turned up to the clinic without the parents. (Paediatrician 15)*

Several clinicians observed it was often the parent/carer who took information on board on behalf of the young person.

*…they all nod and say 'uh huh' but it tends to be the parents who take on that information more. (Child Psychiatrist 08)*

However parents and carers also needed to understand the relevant information themselves. This was referred to by one clinician when describing how much an ADHD nurse could help liaison and information exchange between services.

*I mean one of the most common complaints that I get from parents is that we just don't understand how to deal with our children, we just don't understand how to manage their difficulties when it comes to ADHD. (Paediatrician 05)*
